# Changes in smoke alarm coverage following two fire department home visiting programs: what predicts success?

**DOI:** 10.1186/s40621-014-0030-3

**Published:** 2014-11-24

**Authors:** Andrea C Gielen, Elise C Perry, Wendy C Shields, Eileen McDonald, Shannon Frattaroli, Vanya Jones

**Affiliations:** Johns Hopkins Bloomberg School of Public Health, Johns Hopkins Center for Injury Research and Policy, 624 N. Broadway, Baltimore, 21205 MD USA

**Keywords:** Injury prevention, Smoke alarms, Fires and burns, Home visits, Community health workers, Community intervention

## Abstract

**Background:**

Door-to-door canvassing and installation of smoke alarms have been found to be effective at increasing the number of homes protected. This analysis reports on how smoke alarm coverage changes six months after a home visiting program in a large urban sample, and how this change varies by characteristics of the residents and characteristics of the services delivered during the home visit.

**Methods:**

Fire department Standard and Enhanced home visiting programs were compared. During the home visit, fire fighters installed lithium battery smoke alarms. Residents in the Enhanced program received tailored education about fire safety. Six months after the home visit, participating residences were visited to complete a follow-up survey and to have the installed alarms checked.

**Results:**

81% of the 672 homes that had a working smoke alarm on every level of the home at the end of the home visit remained safe at follow-up, and 87% of the residents found the home visit was very useful, and these rates did not differ between the Enhanced and Standard programs. The degree to which firefighters delivered their services varied, although households in which the resident’s engagement with the fire department team was rated as excellent were 3.96 times as likely to be safe at follow-up compared to those with poor or fair resident engagement (p=0.03).

**Conclusions:**

There is a need to better understand how to maximize the time spent with residents during smoke alarm home visiting programs. This study helps with the development of methods needed for implementing and evaluating such programs in real-world settings.

**Electronic supplementary material:**

The online version of this article (doi:10.1186/s40621-014-0030-3) contains supplementary material, which is available to authorized users.

## Background

Residential fires present a threat to the public’s health and safety. In 2012, there were approximately 365,000 fires occurring in home structures resulting in 2,380 civilian deaths and 12,875 civilian injuries (Karter [2012]). Low-income neighborhoods are at substantially higher risk, in part due to the presence of older and vacant housing that increases fire risk, and high proportions of immigrant populations with limited ability to read and speak English that decreases potential impact of communication about preventive measures (Istre et al. [2001]; Shai [2006]; Schachterle et al. [2012]).

Working smoke alarms reduce the risk of death in the event of a house fire by 50% (Istre et al. [2001]). Having a working smoke alarm with a long-lasting lithium battery on every level of the home is the recommended best practice according to the Centers for Disease Control and Prevention (Ballesteros et al. [2005]). In an observational survey of homes in Baltimore City, 97% had at least one working smoke alarm, but less than half had one on every level (Stone et al. [2007]). An estimated 20-50% of smoke alarms in homes are non-functional, and many residents do not know if their smoke alarms are working (Sidman et al. [2011]; Stepnitz et al. [2012]). The percentage of homes with functioning smoke alarms ranges from 34%-93% among high-risk communities (Liu et al. [2011]).

Door-to-door canvassing and in-home installation of smoke alarms have been found to be the most effective method for increasing the number of homes protected. (Ta et al. [2006]) In a meta-analysis, the most intensive smoke alarm programs, those with a combination of education, low cost or free equipment and direct installations, and those programs that installed lithium battery alarms reported the highest rates of coverage (Cooper et al. [2012]). The prevalence of working smoke alarms after installation has been reported between 79% - 92% at 12 months and 64%-82% at 3-4 years (Ta et al. [2006]; Yang et al. [2008]; Peek-Asa et al. [2010]).

Fire departments and other community organizations that want to increase the prevalence of working smoke alarms in their neighborhoods could benefit from knowing more about how such programs can be most effectively implemented. In this study, the following questions are addressed: 1) How does smoke alarm coverage change six months after a home visiting program in a large urban sample? 2) How does this change in smoke alarm coverage vary by characteristics of the residents and characteristics of the services delivered during the home visit?

## Methods

### Study design

The Johns Hopkins Home Safety Project was a partnership with the Johns Hopkins Center for Injury Research and Policy (JHCIRP), Baltimore City Fire Department (BCFD), Environmental Justice Partnership, and the Urban Health Institute. As previously described, 12 census tracts were selected in East Baltimore – 6 were randomly assigned to receive the Standard BCFD home visiting program and 6 were randomly assigned to receive an Enhanced home visiting program (described below) (Gielen et al. [2013]). From April 2010 to April 2011, a total of 171 canvassing events occurred (82 in the Standard and 89 in the Enhanced areas) during which the firefighters went door-to-door to deliver the home visiting programs. The firefighters came from 21 different fire companies, and were assigned by the BCFD based on the jurisdiction of their fire station and the area to be canvassed. Over the course of the study, the firefighters could have visited homes receiving either the Enhanced or the Standard program, but the distinction was not pointed out to them because the protocol for the firefighters was the same in both. Residents who accepted the home visit and completed both a baseline and six-month follow up survey are the subjects of this analysis, making this a quasi-experimental study design.

### Standard home visiting program

Based on formative work done by the partnership at the outset of the project (Frattaroli et al. [2012]), the BCFD created a specific home visiting protocol that was adopted throughout the Department. Training in the new protocol was provided to all firefighters either through in-person training provided by the Fire Marshal’s Office, or for those firefighters who could not attend the training, the materials were provided for them to review on their own. The protocol included having teams of four BCFD firefighters going door-to-door in their assigned neighborhoods and offering free alarms, which they installed on every level of the home; they also tested CO levels in the home. To provide information and encouragement to maintain working smoke alarms, the firefighters educated the resident about the smoke alarms’ long-lasting batteries, their hush feature, how to use alarm, fire prevention (cooking, electrical, heating issues), fire escape planning, and CO safety. Residents received the smoke alarm instruction manual, and a home safety checklist. The smoke alarms were obtained from the Maryland Department of Health and Mental Hygiene.

### Enhanced home visiting program

Firefighters followed the same protocols for installation and resident education as in the Standard program, with the following additions: 1) community health workers (CHWs) went door-to-door one week in advance of the fire department’s visit and on the day of the visit to inform residents about the free services, answer residents’ questions about services, and encourage residents to participate; 2) a safety educator visited the home with the firefighters to provide tailored home safety education on hot water temperatures and CO poisoning; and 3) a mobile safety center with interactive educational exhibits and low cost safety products parked in the neighborhood on the day of the fire department’s visit, and residents were invited to visit it (Bulzacchelli et al. [2009]; Gielen et al. [2009]).

### Hypotheses and rationale

For this analysis, we hypothesized that smoke alarm coverage at six-month follow-up would be higher among residents in the Enhanced home visiting program compared to those in the Standard home visiting program. The difference between these two programs is that those in the Enhanced program received additional education from a safety educator who accompanied the firefighters, and we assumed that would increase the resident’s motivation to maintain their alarms in good working order. Second, we hypothesized that smoke alarm coverage at the six-month follow-up would be higher when there was greater compliance with the home visit protocol by the firefighters, and when the residents responded more favorably to the visit. This hypothesis assumes that the resident’s motivation to maintain their smoke alarms would be greater when they responded more positively to having the firefighters in their homes, and the better the firefighters were at completing all the installation and education tasks. We had no hypothesis about the relationship between firefighter fidelity to implementing their tasks and resident response because the resident did not know what the firefighter protocols involved, although we do examine those potential relationships.

### Data collection

The Johns Hopkins Institutional Review Board approved this study. Data collection took place inside residents’ homes at the time of the home visit (baseline) and six months later (follow-up). Once permission to enter the home was given for the baseline home visit, the firefighters installed the smoke alarms while trained data collectors recorded their activities (number and location of alarms, fidelity to protocol, etc.). When the firefighters finished installing the alarms, the data collectors asked the resident to complete a brief survey about their home visit experience and their home safety knowledge (the baseline survey). If residents declined this baseline survey, they were not offered a follow-up visit. If residents completed the baseline survey, they were informed about the six-month follow-up and asked if they would be willing to participate. Over the course of the study 49 data collectors were employed, all of whom were trained in the data collection protocols through an in-office training program followed by two in-the-field sessions in which they shadowed a veteran data collector. They were assigned to the home visits based on their availability, and could collect data in both the Standard and Enhanced groups, where the fire department protocols were identical.

In the Standard program, the visit ended after residents completed the baseline survey. In the Enhanced program, responses to the baseline survey were used by the safety educator to provide specific educational messages tailored to the responses (McDonald et al. [2013]). The extra education on carbon monoxide and hot water scald burns provided by the safety educator was the main difference in the home visit experience between the Enhanced and Standard programs. The mobile safety center was not able to attend all of the canvassing events and there was insufficient uptake of it when it was present, so no further data from that component of the program is considered here.

Six months after the home visit, each participating residence was visited to complete the follow-up survey and to have all the installed alarms checked. Homes were visited up to ten times on nonconsecutive days during daylight hours until the resident completed or refused the follow-up or they were deemed ineligible. Respondents were ineligible if the original respondent had moved, the home was vacant, or the respondent was impaired and unable to complete the follow up visit. The remainder who were lost to follow-up were those whose survey windows expired (ten attempts by data collectors to reach the participant and complete the survey).

### Measures

#### Sociodemographic characteristics of residents

Sociodemographic information was collected during the follow-up interview. Residents self-reported information about household size, composition, and income; respondent age, education, employment, and ethnicity.

#### Characteristics of the home visit

##### Implementation fidelity (smoke alarm, education)

During the home visit, data collectors observed fire department activities and recorded fidelity to the fire department’s protocol using a process evaluation checklist. The checklist covered both smoke alarm installation and safety education expectations for fire personnel.

The smoke alarm section had five tasks the fire department personnel were to complete: 1) tell resident about the long-lasting battery; 2) describe the alarm’s hush feature to the resident; 3) demonstrate how to use alarm; 4) give the instruction manual to the resident; and 5) install the alarms using screws. The data collector recorded a yes/no for each item, which resulted in a score of 0-5 on the smoke alarm implementation fidelity score.

The safety education section included six tasks: 1) discuss CO safety; 2) discuss fire escape planning; 3) discuss cooking safety; 4) discuss electrical safety; 5) discuss heating safety; and 6) distribute a home safety checklist that reinforced this information. The data collector recorded a yes/no for each item, which resulted in a score of 0-6 on the education implementation fidelity score.

##### Observer reported ratings at the home visit (Fire Department, Resident Engagement)

At the end of the home visit, data collectors provided their subjective assessment of the home visit by rating the overall quality of the visit and the residents’ engagement with the fire department personnel (poor, fair, good, very good, or excellent). Based on the distribution of responses, the categories were recoded for analysis with “poor” and “fair” combined.

##### Resident reported ratings at follow-up

During the follow up, interviewers asked residents to rate how useful the home visit was to them (not at all, just a little, somewhat or very useful). Based on the distribution of responses, the categories were recoded for analysis with “not at all” and “just a little” combined.

#### Observed safety behaviors

During the original home visit, data collectors recorded the number and location of all smoke alarms installed by the BCFD. During the follow up visit, data collectors recorded the location of each of the installed alarms and tested them by pushing the test button. A summary variable was created to indicate whether there was a working smoke alarm (of any type) on every level of the home at baseline and at follow-up. At both time points, a home was considered to have “all levels safe” if there was a working smoke alarm (of any type) on every level of the home. At baseline homes were coded as “not having all levels safe” if the firefighter did not install an alarm on every level (most often because the resident did not allow access to a level); and, at follow up, if the installed alarms were either missing or non-functioning.

### Statistical analysis

Demographic characteristics of the study sample were tabulated and compared between the enhanced and standard study conditions. A paired t-test was used to compare changes in smoke alarm coverage in the two study groups. The two study groups were compared on receipt of the home visit components and resident reactions, using rank sum statistics. Rank sum tests were used because the outcome has ordinal response categories. Finally, multiple logistic regression was used to identify correlates of having working smoke alarms on all levels at the six-month follow-up, including covariates on which the two study groups differed, smoke alarm status at baseline, and variables that were significant in bivariate analyses (data not shown). Number of people in the home was excluded from the model because of its correlation with having children in the home.

## Results

### Sample

**Figure 1:**
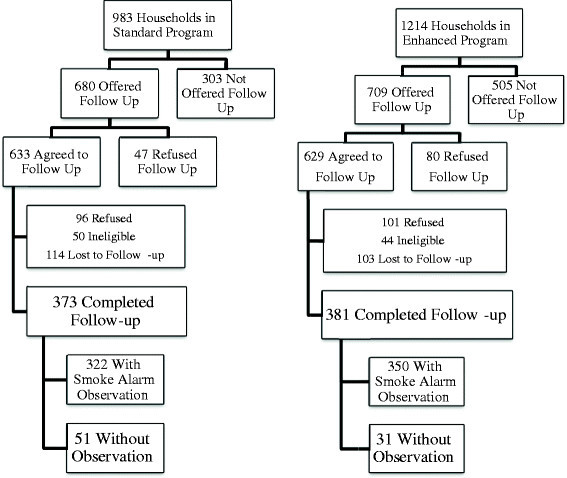
**Study households participation flow chart.**

As shown in Figure 1, 983 residents in the standard program and 1214 in the enhanced program areas participated in the original home visits. Of these, 680 (69.18%) in the standard program and 709 (58.40%) in the enhanced program agreed to complete the baseline survey (p < 0.01), which made them eligible to recruit for the six-month follow-up. Of those eligible for the follow-up, 633 (93.08%) and 629 (88.72%) accepted in the standard and enhanced areas respectively (p < 0.01). Between January 2011 and December 2011, 754 follow-up interviews were completed (381 in the enhanced and 373 in the standard communities).

There was no difference in the completion rates across groups for the follow-up survey: 373 (58.92%) in the standard area and 381 (60.57%) in the enhanced (p = 0.55) completed the follow-up. Ninety-six (15.16%) people in the standard area and 101 (16.06%) in the enhanced area refused the follow up survey; 50 (7.90%) in the standard area and 44 (7.00%) in the enhanced area were ineligible.

Follow-up participants in the Standard program were more likely to be older, and to have children and a larger number of people living in the home relative to households in the Enhanced program (Table 1).

**Table 1:** **Sociodemographic characteristics of follow-up participants: comparing the standard and enhanced programs**

		Standard N =341 (%)	Enhanced N =367 (%)	Total N = 708 ^1^ (%)	Chi-square
**Gender**	**Male**	82 (24.05)	110 (29.97)	192 (27.12)	3.14 (p = 0.08)
	**Female**	259 (75.95)	257 (70.03)	516 (72.88)	
**Age**	**18 to 24**	13 (3.82)	14 (3.84)	27 (3.83)	12.89 (p = 0.01)
	**25 to 34**	85 (25.00)	60 (16.44)	145 (20.57)	
	**35 to 44**	58 (17.06)	74 (20.27)	132 (18.72)	
	**45 to 55**	80 (23.53)	72 (19.73)	152 (21.56)	
	**55 and above**	104 (30.59)	145 (39.73)	249 (35.32)	
**Household Role**	**Head of Household**	289 (84.75)	302 (82.74)	591 (83.71)	0.52 (p = 0.50)
	**Other**	52 (15.25)	63 (17.26)	115 (16.29)	
**Education**	**< high school diploma**	59 (17.35)	76 (20.82)	135 (19.15)	3.80 (p = 0.28)
	**HS diploma/GED**	128 (37.65)	145 (39.73)	273 (38.72)	
	**Some college**	67 (19.71)	54 (14.79)	121 (17.16)	
	**Completed college**	86 (25.29)	90 (24.66)	176 (24.96)	
**Household income below the poverty line?**	**Yes**	75 (26.69)	83 (27.21)	158 (26.96)	0.02 (p = 0.89)
	**No**	206 (73.31)	222 (72.79)	428 (73.04)	
**Homeowner Status**	**Rent**	144 (42.73)	148 (40.66)	292 (41.65)	0.31 (p = 0.58)
	**Own or pay mortgage**	193 (57.27)	216 (59.34)	409 (58.35)	
**Children in home (<18 y)**	**Yes**	180 (52.79)	137 (37.33)	317 (44.77)	17.07 (p < 0.01)
	**No**	161 (47.21)	230 (62.67)	391 (55.23)	
**Number of people in the home**	**1 person**	34 (10.00)	77 (21.10)	111 (15.74)	18.01 (p < 0.01)
	**2-3 people**	169 (49.71)	174 (47.67)	343 (48.65)	
	**4-6 people**	119 (35.00)	98 (26.85)	217 (30.78)	
	**7 or more people**	18 (5.29)	16 (4.38)	34 (4.82)	

^1^46 residents did not respond to survey questions; some variables do not add up to 708 due to missing item responses.

### Smoke alarm changes from baseline home visit to 6-month follow-up

In the Standard program 288 (89.44%) homes had all levels safe after the home visit (baseline) compared to 324 (92.57%) homes in the Enhanced program. (Table 2) In the Standard program homes at follow-up, 235 (72.98%) had all levels safe compared to 274 (78.28%) in the Enhanced program homes.

**Table 2:** **Smoke alarm coverage at baseline and follow-up: comparing the standard and enhanced programs** ^**1**^

All levels safe? ^2^		Standard program			Enhanced program			Total sample		
		Follow up (N = 322)			Follow up (N = 350)			Follow up (N = 672) ^3^		
		Yes	No	Total	Yes	No	Total	Yes	No	Total
**Baseline**	**Yes**	226 (78.47%)	62 (21.53%)	288 (100%)	269 (83.02%)	55 (16.97%)	324 (100%)	495 (80.88%)	117 (19.12%)	612 (100%)
	**No**	9 (26.47%)	25 (73.53%)	34 (100%)	5 (19.23%)	21 (80.77%)	26 (100%)	14 (20.33%)	46 (76.67%)	60 (100%)
**Total**		235 (72.98%)	87 (27.02%)	322 (100%)	274 (78.28%)	76 (21.71%)	350 (100%)	509 (75.74%)	163 (24.26%)	672 (100%)

^1^Paired t-test for changes from baseline to follow-up between Standard versus Enhanced: t = 0.68 (p = 0.50).

^2^All Levels Safe was defined as working long life battery operated or hard-wired alarms on all levels of the home.

^3^Smoke alarm status at follow up was unavailable for 51 households in the standard and 31 households in the enhanced area.

Table 2 examines the proportion of homes with all levels safe at follow-up as a function of their status at baseline. For the entire sample, 80.88% of homes that were safe at the end of the home visit remained safe at follow-up. In the Standard program homes, 78.47% that were safe at baseline were safe at the six month follow-up, compared to 83.02% of homes in the Enhanced program. Of the homes that did not have all levels safe at the end of the home visit, 26.47% of those in the Standard and 19.23% of those in the Enhanced were safe at the six-month follow-up, which could occur, for example if the resident added a new alarm or replaced a battery where it was needed. The changes from baseline to follow up between the Enhanced and Standard programs were not statistically significantly different (t = 0.68, p = 0.50).

### Predictors of smoke alarm coverage at 6-month follow-up

Table 3 compares characteristics of program implementation between the Standard and Enhanced programs. There was no difference in the fire department’s Smoke Alarm fidelity score, with an overall average of 59.51% of the tasks completed across all the home visits. For the Education fidelity score, 23.72% of the tasks were completed on average in the Enhanced program compared to 19.91% in the Standard program (p = .05). Overall, the group receiving the Enhanced program had higher ratings on the delivery of services (p < 0.01), and for the resident’s engagement, the difference approached statistical significance (p = 0.10).

**Table 3:** **Ratings in-home intervention components by observers and residents: comparing standard and enhanced study programs**

Observer ratings at baseline home visit		Standard N = 373	Enhanced N = 381	Total N = 754 ^1^	Test statistic
		Mean % (SD)	Mean% (SD)	Mean% (SD)	
**Fire Department Implementation**	Smoke Alarm Fidelity^2^	57.97 (22.26)	60.84(22.25)	59.51 (23.93)	Rank Sum (p = 0.16)
	Education Fidelity^3^	19.91 (20.02)	23.72(23.14)	21.85 (21.73)	Rank Sum (p = 0.05)
		**N (%)**	**N (%)**	**N (%)**	
**How would you rate the FD’s delivery of services?**	Poor or Fair	108 (29.59)	93 (24.67)	201 (27.09)	Rank Sum (p = <0.01)
	Good	151 (41.37)	137 (36.34)	288 (38.81)	
	Very Good	86 (23.56)	100 (26.53)	186 (25.07)	
	Excellent	20 (5.48)	47 (12.47)	67 (9.03)	
**How would you rate the resident’s engagement with the FD team?**	Poor or Fair	46 (12.57)	21 (5.57)	67 (9.02)	Rank Sum (p = 0.10)
	Good	157 (42.90)	182 (48.28)	339 (45.62)	
	Very Good	128 (34.97)	127 (33.69)	255 (34.32)	
	Excellent	35 (9.56)	47 (12.47)	82 (11.04)	
**Resident ratings at follow-up**		**Standard N = 311**	**Enhanced N = 341**	**Total N = 652** ^**4**^	
**Thinking back to the home visit, how useful would you say it was for you?**	Very useful	266 (86.08)	295 (87.28)	561 (86.71)	Rank Sum (p = 0.61)
	Somewhat useful	33 (10.68)	37 (10.95)	70 (10.82)	
	Just a little or not at all	10 (3.24)	6 (1.78)	16 (2.47)	

^1^Some variables do not add up to 754 due to missing item responses.

^2^Based on 5 components (tell resident about 10 year battery, show hush feature, show how to use alarm, give instruction manual to resident, screw in alarms).

^3^Based on 6 components (distributed checklist, discuss CO safety, discuss escape plan, discuss cooking safety, discuss electrical safety, discuss heating safety).

^4^Of the 708 residents completing the follow up survey, 652 recalled the fire department home visit and answered this question.

There was no difference between the Standard and Enhanced programs in the residents’ rating of the usefulness of the program (p = 0.61), with 86.71% rating it as very useful (Table 3). In a separate bivariate analysis, Smoke Alarm fidelity, but not Education fidelity, was associated with the resident’s usefulness rating of the home visit (p = 0.01). Participants reporting the home visit was “Very Useful” had the highest mean Smoke alarm fidelity (58.36%), compared to those who responded the home visit was “Somewhat Useful” (48.64%) and “Not at all useful” (40.00%).

Of all of these program implementation variables, only the observer’s rating of the resident’s engagement with the firefighters was significantly related to the outcome of having all levels safe at follow-up in the bivariate analyses (p < 0.01). The proportions that were safe vs. unsafe were 14.86% vs. 85.14% among those rated “Excellent”; 21.65% vs 78.35% among those rated “Very Good”; 25.74% vs 74.26% among those rated “Good”; and 28.81% vs 71.19% among those rated as “Fair or Poor”.

In the multiple logistic regression model predicting “all levels safe” at follow-up (Table 4), study group was not significant. Regardless of study group, if the home was safe (i.e., had working alarms on all levels) at the end of the original home visit, it was significantly more likely to be safe at follow up (OR =17.06, p < 0.01) compared to homes that were unsafe at the end of the home visit. Survey respondents who were 55 and older were 3.61 times as likely to be in a home that was safe at follow up relative to 18 to 24 year olds (p = 0.01). Controlling for age and the other variables in the model, households in which respondents’ engagement with the fire department team was rated as excellent were 3.96 times as likely to have all levels safe compared to those households rated as poor or fair engagement with the program (p = 0.03). There was no interaction between age and resident engagement (data not shown).

**Table 4:** **Smoke alarm coverage six months after home installation program: multiple logistic regression model of all levels of the home safe at follow-up** **, N = 626**

		All Levels Safe at Follow-up	p-value
**Program**	Standard	Reference	
	Enhanced	1.08	0.72
**Smoke Alarm Safety at Baseline**	Not Safe	Reference	
	Safe	17.06	<0.01
**Age of Respondent**	18 to 24	Reference	
	25 to 34	1.87	0.50
	35 to 44	2.93	0.19
	45 to 54	2.31	0.73
	55 and above	3.61	0.01
**Household Role**	Head of Household	Reference	
	Other	0.63	0.09
**Children in home?**	Yes	Reference	
	No	1.07	0.77
**How would you rate the resident’s engagement with the FD team?**	Poor or Fair	Reference	
	Good	1.78	0.54
	Very Good	2.22	0.57
	Excellent	3.96	0.03

^1^All levels safe defined as working long lasting lithium battery alarms or hard wired alarms on all levels of the home.

## Discussion

Our analysis did not support the hypothesis that smoke alarm coverage at the six-month follow-up would be higher in the enhanced program because they received additional education from a safety educator who accompanied the firefighters. Overall, a high proportion of homes that had working smoke alarms on all levels at the end of the home visit installation maintained these alarms (81%). This may be partially explained by the fact that tamper resistant, long-lasting lithium battery alarms with a hush feature were installed, presumably making it less likely that the batteries would be removed or fail during the six-month interval (Jackson et al. [2010]; Yang et al. [2008]: Peek-Asa et al. [2010]; Cooper et al. [2012]).

Our results only partially supported the second hypothesis concerning compliance with the home visit protocol by the firefighters and resident response to the visit. Those residents who were rated as more engaged with the fire department team were significantly more likely to have working alarms on all levels at follow-up, even when controlling for standard vs. enhanced program, smoke alarm status at baseline, and multiple demographic variables. However, no relationship between the firefighters’ fidelity to the home visit protocol and having working smoke alarms at follow-up was observed, which may be due to the overall low levels of fidelity to the smoke alarm and education protocols, and the minimal amount of effort needed on the part of the resident to maintain the alarms. Why rates of fidelity to the smoke alarm and education protocols was low warrants further exploration, as do our findings related to the lack of a relationship between other elements of program implementation, resident ratings of the visit usefulness and program outcomes. Further research is needed to better understand the “active ingredients” of the home visit that engage the resident as well as other home visit outcomes such as safety knowledge.

The selectivity of the sample is a limitation. In a previous analysis (Gielen, et al. 2013), we showed that our standard and enhanced neighborhoods were comparable to each other on key variables (e.g., income below poverty, education), but were poorer and less well educated than Baltimore City as a whole. The sample analyzed here was drawn from those residents who were home on the day of the canvassing, and who let the fire department into their homes. While the fire department provided smoke alarms to all residents who let them in, we could only collect evaluation data from those who agreed to complete our data collection at the home visit and at follow up. Comparing our final sample to Baltimore City as a whole found that they maybe slightly more disadvantaged on income below the poverty line (26.96% vs 20.00%), and less so on completing high school (80.85% vs 76.90%) (Gielen et al. 2012).

Our recruitment strategy resulted in residents having multiple opportunities to decline participation. Most refusals occurred at the time of the home visit when residents were asked to complete the baseline survey (a prerequisite for being offered the follow-up), perhaps because there was already so much activity and several people in the home. The slightly longer home visit experience for those in the enhanced program may have also contributed to higher refusals for follow-up in that group. Residents could decline the follow-up both when it was offered after the baseline or when it was attempted, and some follow-up visits were not completed due to non-working telephones, moving, and lack of response after multiple attempts. Thus, the sample is self-selected, and our rates of smoke alarm maintenance may not be representative of the larger population in the sampled census tracts or in the City overall.

Our follow-up duration of six months is consistent with some previous studies, but not as long as would be ideal. Nevertheless, our rate of 81% is within the previously reported ranges across multiple follow-up periods (Ta et al. [2006]; Yang et al. [2008]; Peek-Asa et al. [2010]). This result, combined with the finding that the vast majority of residents (86.71%) reported that the home visit was very useful bodes well for long-term impact of fire department home visiting programs on fire deaths and injuries. A remaining challenge is to better understand whether and to what extent the educational component of a home visit can make a difference in preventing fires and other home injuries in the first place.

## Conclusions

There is now a robust literature supporting the benefits of comprehensive smoke alarm installation programs in many types of populations, including rural and urban areas (Cooper et al. [2012]; Liu et al. [2011]; Ta et al. [2006]; Yang et al. [2008]; Peek-Asa et al. [2010]; Gielen et al. [2013]). We found that the vast majority of residents rated the home visit as very useful, and our measure of resident engagement with the firefighters during the home visit was significantly associated with having the recommended number of working smoke alarms at follow-up. However, our observers noted considerable variability in the degree to which the firefighters delivered their services, and little is known about firefighters’ perspectives and the barriers and facilitators they face in adding home visiting to their skill set and work load (Frattaroli et al. [2012]). Thus, it is time to better understand how to maximize the time spent with residents, and this study helps with the development of methods needed for implementing and evaluating home visiting programs in real-world settings.

## Authors’ original submitted files for images

Below are the links to the authors’ original submitted files for images. Authors’ original file for figure 1
