# Supplementary figures and images for: Changes in smoke alarm coverage following two fire department home visiting programs: what predicts success?
Source: Inj Epidemiol. 2014 Nov 24;1(1):30. doi: 10.1186/s40621-014-0030-3 (PMC5005669; doi:10.1186/s40621-014-0030-3)

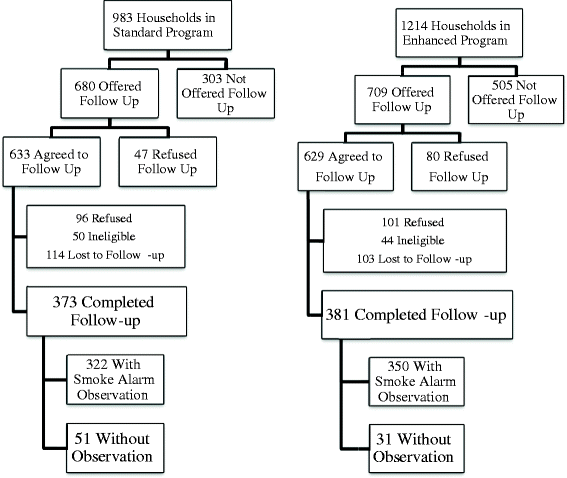

Supplement: Supplementary file 1 — Authors’ original file for figure 1 [file 40621_2014_30_MOESM1_ESM.gif]
